# Supplementary material for: Genome-wide association study of Idiopathic Pulmonary Fibrosis susceptibility using clinically-curated European-ancestry datasets
Source: medRxiv. 2025 Feb 2:2025.01.30.25321017. Preprint. [Version 1] doi: 10.1101/2025.01.30.25321017 (PMC11838657; doi:10.1101/2025.01.30.25321017)
Supplement: Supplement 1 [file media-1.pdf]

SUPPLEMENTAL MATERIAL

Genome-wide association study of Idiopathic Pulmonary Fibrosis susceptibility using clinically-curated European-ancestry datasets

Index

SUPPLEMENTAL METHODS 3

Study samples 3

Statistical analysis 4

Functional annotation 5

SUPPLEMENTAL TABLES 6

Table S1. Sample size of the study. 6

Table S2. Bayesian fine mapping results from new signals sorted by posterior inclusion probability. 7

Table S3. Functional assessment of sentinels and variants with the posterior inclusion probability (PIP)>0.1 in the credible set. 8

Table S4. Association results for previously reported genome-wide signals. 9

Table S5. Colocalisation analysis between the IPF risk signals and the gene expression eQTL signals in lung, fibroblasts and blood. 10

SUPPLEMENTAL FIGURES 11

Figure S1. Quantile-quantile plot 11

Figure S2. Mirror plots of the signals of interest 12

REFERENCES 13

SUPPLEMENTAL METHODS

Study samples

We conducted an analysis from seven independent case-control studies of idiopathic pulmonary fibrosis (IPF) previously documented. For all seven studies, IPF cases were diagnosed based on the guidelines established by the American Thoracic Society and European Respiratory Society (1)(2)(3). The seven datasets are composed by Colorado (4), Chicago (5), UK (6), IPF Job Exposures Study (IPF-JES) (7), Genentech (8) (9), United States, United Kingdom, and Spain (UUS) (10), and Study of Clinical Efficacy of Antimicrobial Therapy Strategy Using Pragmatic Design in Idiopathic Pulmonary Fibrosis - University of California, Davis (CleanUP-UCD) (11).

The Colorado study (4), a cohort of 1,515 patients with fibrotic idiopathic interstitial pneumonias (fIIP), was sourced from various US cohorts (including National Jewish Health IIP population, InterMune IPF trials, UCSF, Vanderbilt University IIP population, and the National Heart Lung and Blood Institute Lung Tissue Research Consortium). These cases were matched with 2,455 population controls chosen for their genetic similarity. Genotyping for all individuals was conducted using the Illumina Human 660W Quad BeadChip array.

The US study (previously referred as "Chicago" study) (5) encompassed 541 IPF cases from the University of Chicago, University of Pittsburgh, and the COMET study. It also included 542 population controls from the database of genotypes and phenotypes (dbGaP) and the University of Pittsburgh. Genotyping for both cases and controls was carried out using the Affymetrix Genome-Wide Human SNP 6.0 array.

The UK study (6) gathered a total of 612 IPF cases from 9 different centres across the UK, and 3,366 matching controls selected from the UK Biobank. Cases were genotyped with the Affymetrix UK BILEVE array, while controls were genotyped with the UK Biobank array.

The Idiopathic Pulmonary Fibrosis Job Exposures Study (IPF-JES) (7) included 416 men from England, Scotland, or Wales diagnosed with IPF, along with 2,465 matched male controls recruited to the IPF-JES study or selected from UK Biobank. Genotyping was carried out using the Affymetrix UK Biobank array.

The Genentech study (8) (9) comprised 813 cases derived from three IPF clinical trials (ASCEND, CAPACITY, and RIFF) and 3,949 controls from four non-IPF clinical trials including age-related macular degeneration, diabetic macular oedema, multiple sclerosis, asthma, and inflammatory bowel disease. Genotyping was accomplished through whole-genome sequencing using the HiSeq X Ten platform (Illumina) with an average read depth of 30X. Individuals were filtered with ADMIXTURE v1.23 to keep only the European ancestry individuals, and also by missingness, relatedness and heterozygosity. Variants were filtered according to missingness >0.1, Hardy-Weinberg Equilibrium (HWE) (p=5x10^{-8}), case-control missingness and allele balance.

The UUS study (United States, United Kingdom, and Spain) (10) included 793 IPF cases from 7 study cohorts (ACE, PANTHER, UCD, Chicago, UCSF, PROFILE, and Spain), along with 9,999 population controls selected from the UK Biobank to match ancestry, sex, and smoking

distribution. Genotyping for cases was done using the Affymetrix UK Biobank and Spanish Biobank arrays, while controls were genotyped with the UK Biobank array.

The CleanUP-UCD study (11), encompassed a total of 469 IPF cases drawn from a randomized clinical trial conducted across 35 locations in the United States, and 32 participants were recruited at University of California Davis (UCD). Additionally, 2,455 population controls were selected from the UK Biobank. Genotyping was performed on both cases and controls using the Affymetrix UK Biobank array.

Six case-control studies (Colorado, IPF-JES, UK, US, UUS, and CleanUP-UCD) were imputed using the TOPMed WGS reference panel (GRCh38) via the TOPMed Imputation Server, ensuring that only SNPs available in both (cases and controls) were included.

For the X chromosome, we performed the analysis of four independent case-control studies, including Colorado, UK, UUS and CleanUP-UCD. Genotyping and quality control were performed as previously described (following previously described methods: HWE, CR95% and MAF) and imputed using TOPMed. We performed association analysis adjusted for 10 principal components using PLINK v2 (12) with the option --xchr-model 2, to consider the males and female dosages on a 0-2 scale. Variants with a poor imputation quality ($r2<0.5$) and a low minor allele frequency ($MAF<0.01$) were removed. We meta-analysed results using inverse-variance weighted fixed effect meta-analysis using METAL (13) and excluded SNPs that were not measured in at least two studies. We estimated the genomic inflation factor using the classical lambda and corrected by this value.

Statistical analysis

Bayesian fine-mapping

Fine mapping was used to generate a set of genetic variants with a 95% probability of containing the causal variant associated with a given genetic signal (95% credible set). This process was performed using R version 4.3.1. The fine-mapping strategy used, using the Wakefield approximate Bayes factor (14), assumed the existence of a single measured causal variant. The variants from the credible set were annotated using ANNOVAR v07.06.20 (15).

Colocalisation

For those variants showing associations with gene expression levels, colocalisation analyses were performed in lung, cultured fibroblasts, and whole blood tissues (Genotype-Tissue Expression Project: GTEx) corresponding to the relevant genes. These analyses were performed using the coloc (16) package in R version 4.3.1.

Colocalisation analyses were used to determine whether the same causal variant was associated with both IPF susceptibility and gene expression in GTEx. The posterior probability was estimated using the approximate Bayes factor:

- H0: neither IPF susceptibility nor gene expression have a genetic association in that region
- H1: only IPF susceptibility has a genetic association in that region
- H2: only gene expression has a genetic association in that region
- H3: both IPF susceptibility and gene expression are associated, but with different causal variants

- H4: both IPF susceptibility and gene expression are associated and share a single causal variant

If the posterior probability in favour of the alternative hypothesis, indicating the presence of a common single causal variant for both IPF susceptibility and gene expression (H4), exceeded 70%, we inferred that the IPF and gene expression signals showed colocalisation.

Functional annotation

We conducted a thorough functional impact assessment using empirical data from several integrated software tools and datasets. We used RegulomeDB (17) and The Open Targets Post-GWAS portal (18) to assess the regulatory potential and prioritise the functional roles of each SNP and its associated gene. In addition, we used Open Targets (https://www.opentargets.org/) (18) to access cross-referencing of genetic variants with a wide range of phenotypes. Variants were annotated using various databases for variant type, protein function and pathogenic potential using Ensembl VEP v105.

Active chromatin marks

We also functionally annotated the highlighted regions based on active chromatin marks, including DNase I hypersensitivity hotspots (DHS), open chromatin peaks, transcription factor footprints, enhancer RNAs (eRNAs), Assay for Transposase-Accessible Chromatin using sequencing (ATACseq), ChIP sequencing (CHIPseq), and epigenetic modification of the DNA packaging protein histones (H3K27ac) (19).

Mouse-knockout orthologs with a respiratory phenotype

We selected human orthologs of mouse knockout genes with respiratory phenotypes listed in the International Mouse Phenotyping Consortium (https://www.mousephenotype.org/), within ±500 kb of a IPF sentinel variant.

SUPPLEMENTAL TABLES

Table S1. Sample size of the study.

	Cases	Controls	Autosomal variants analysed	X chromosome variants analysed
Colorado	1,515	4,683	24,275,956	238,835
US	541	542	13,539,842	---
UK	612	3,365	19,550,111	262,880
IPF-JES	416	2,465	18,681,239	---
Genentech	813	3,949	14,955,256	---
UUS	793	9,999	33,838,128	255,656
CleanUP-UCD	469	2,455	19,500,953	262,831
Total	5,159	27,458	25,290,839	262,089
The number of individuals and variants shown is the result after quality controls.				

Table S2. Bayesian fine mapping results from new signals sorted by posterior inclusion probability.

CHR	BP	rsID	Location	Nearest genes	A1*/A2	A1 Freq.	OR (95%)	P	PIP (95%)
1	155179278	rs9426886	intronic	*TRIM46*	A/T	0.568	0.861 (0.820-0.904)	$1.53x10^{-9}$	0.208
1	155179017	rs11264341	intronic	*TRIM46*	T/C	0.432	1.161 (1.106-1.219)	$1.70x10^{-9}$	0.189
1	155204315	rs2075571	intronic	*THBS3*	T/C	0.428	1.161 (1.106-1.219)	$1.83x10^{-9}$	0.176
1	155183255	rs4971100	intronic	*TRIM46*	A/G	0.430	1.160 (1.105-1.218)	$2.17x10^{-9}$	0.149
1	155185239	rs2070803	downstream	*MUC1\TRIM46*	A/G	0.567	0.864 (0.823-0.907)	$3.74x10^{-9}$	0.089
1	155199564	rs423144	intronic	*THBS3*	T/G	0.429	1.157 (1.103-1.215)	$3.74x10^{-9}$	0.089
1	155185159	rs4971101	downstream	*MUC1\TRIM46*	A/G	0.432	1.156 (1.101-1.214)	$4.77x10^{-9}$	0.070
3	14270502	rs112271207	intergenic	*LSM3\LINC01267*	T/C	0.060	1.356 (1.231-1.494)	$2.23x10^{-11}$	0.691
3	14269725	rs114455129	intergenic	*LSM3\LINC01267*	T/C	0.051	0.743 (0.676-0.817)	$1.36x10^{-10}$	0.118
3	14301732	rs116678873	intergenic	*LSM3\LINC01267*	A/G	0.058	0.744 (0.677-0.818)	$6.81x10^{-10}$	0.029
3	14298964	rs111405596	intergenic	*LSM3\LINC01267*	T/C	0.939	1.335 (1.216-1.465)	$8.45x10^{-10}$	0.024
3	14303378	rs112304124	intergenic	*LSM3\LINC01267*	A/G	0.939	1.342 (1.221-1.476)	$1.10x10^{-9}$	0.019
3	14308107	rs112255370	intergenic	*LSM3\LINC01267*	T/C	0.064	1.334 (1.215-1.464)	$1.14x10^{-9}$	0.019
3	14304955	rs113247615	intergenic	*LSM3\LINC01267*	A/C	0.061	0.745 (0.677-0.819)	$1.17x10^{-9}$	0.018
3	14308058	rs111345960	intergenic	*LSM3\LINC01267*	T/C	0.064	0.811 (0.754-0.872)	$1.24x10^{-9}$	0.017
3	14305318	rs111275427	intergenic	*LSM3\LINC01267*	T/C	0.939	1.233 (1.147-1.326)	$1.24x10^{-9}$	0.017
12	95866696	rs7957346	3'UTR	*SNRPF*	A/C	0.582	0.811 (0.753-0.872)	$1.56x10^{-8}$	0.376
12	95869659	rs71443541	intronic	*CCDC38*	A/G	0.583	0.870 (0.829-0.914)	$2.35x10^{-8}$	0.254
12	95884368	rs4762250	intronic	*CCDC38*	A/G	0.435	1.145 (1.091-1.203)	$3.70x10^{-8}$	0.164
12	95860750	rs6538677	intronic	*SNRPF*	A/G	0.398	1.145 (1.091-1.203)	$5.15x10^{-8}$	0.121
12	95837440	rs76152842	ncRNA_intronic	*LOC105369921*	T/C	0.608	0.876 (0.834-0.920)	$1.06x10^{-7}$	0.063

*A1: effect allele; OR: Odds Ratio; PIP: posterior inclusion probability; Coordinates in GRCh38, Freq.: frequency.

Table S3. Functional assessment of sentinels and variants with the posterior inclusion probability (PIP)>0.1 in the credible set.

Region (PIP>0.1)	Nereast genes	rsID	Location	V2G	CADD	LoFtool	Colocalisation eQTL (GTEx) [highest PP-H4]	RegulomeDB / score	PheWAS ($p<5X10^{-8}$) [Or most significant]	Mouse KO	Active mark annotation	eRNA or footprints
1q22 (0.208)	*TRIM46*	rs9426886	intronic	*THBS3* (sQTL; eQTL)	4.64	0.022	Coloc=61.6% in whole blood (*MUC1*);	eQTL/caQTL + TF binding / chromatin accessibility peak (0.66703)	Hematocrit measurement (p=4.7x10^{-41})	None	DHS; ATACseq; H3K27ac	No
1q22 (0.189)	*TRIM46*	rs11264341	intronic	*ADAM15* (sQTL; eQTL; DHS-promoter correlation)	3.04	0.022		eQTL/caQTL + TF binding / chromatin accessibility peak (0.55436)	Urate levels measurement (p=1.5x10^{-101})	None	DHS; ATACseq; H3K27ac	No
1q22 (0.176)	*TRIM46*	rs4971100	intronic	*THBS3* (sQTL; eQTL)	3.33	None		eQTL/caQTL + TF binding / chromatin accessibility peak (0.55436)	Hematocrit measurement (p=1.2x10^{-40})	None	H3K27ac	No
1q22 (0.149)	*THBS3*	rs2075571	intronic	*THBS3* (sQTL; eQTL)	3.01	0.337		eQTL/caQTL + TF binding / chromatin accessibility peak (0.66703)	Hematocrit measurement (p=6.8x10^{-41})	None	DHS; H3K27ac	No
3p25.1 (0.691)	*LSM3\ LINC01267*	rs112271207	intergenic	*SLC6A6* (PCHi-C)	7.51	None	No significant eQTLs	TF binding or chromatin accessibility peak (0.13454)	Lung function (FEV1/FVC) measurement (p=6.1x10^{-8})	None	ATACseq; H3K27ac	No
3p25.1 (0.118)	*LSM3\ LINC01267*	rs114455129	intergenic	*SLC6A6* (PCHi-C)	0.195	None		TF binding + chromatin accessibility peak (0.609069)	Lung function (FEV1/FVC) measurement (p=1.5x10^{-5})	None	ATACseq; H3K27ac	No
12q23.1 (0.376)	*SNRPF*	rs7957346	3'UTR	*NTN4* (sQTL)	2.9	None	Coloc=64.9% in lung (*NTN4*)	eQTL/caQTL + TF binding / chromatin accessibility peak (0.55324)	Lung function (FEV1/FVC) measurement (p=7.8x10^{-45})	None	ATACseq; H3K27ac	No
12q23.1 (0.254)	*SNRPF*	rs6538677	intronic	*NTN4* (sQTL)	0.03	None		eQTL/caQTL + TF binding / chromatin accessibility peak (0.55324)	Lung function (FEV1/FVC) measurement (p=2.1x10^{-38})	None	ATACseq; H3K27ac	No
12q23.1 (0.164)	*CCDC38*	rs10735335	intronic	*NTN4* (sQTL)	1.66	0.974		eQTL/caQTL + TF binding / chromatin accessibility peak (0.68)	Lung function (FEV1/FVC) measurement (p=2.2x10^{-35})	None	DHS; ATACseq; H3K27ac	No
12q23.1 (0.121)	*CCDC38*	rs4762250	intronic	*NTN4* (sQTL)	11.4	0.974		eQTL/caQTL + TF binding / chromatin accessibility peak (0.55324)	Lung function (FEV1/FVC) measurement (p=6.9x10^{-33})	None	H3K27ac	No

CADD: Combined annotation dependent depletion; DHS: DNase I sensitivity quantitative trait loci; eQTL: Expression quantitative trait loci; eRNA: enhancer RNAO: Knockout; LoFtool: Gene intolerance score based on loss-of-function variants, LoFtool score < 0.1 low tolerance to loss function; PheWAS: Related traits from Open Target Genetics; PP-H4; posterior probability; RegulomeDB model score is ranging from 0 to 1, with 1 being most likely to be a regulatory variant; sQTL: Splicing quantitative trait loci; V2G: Automated variant to gene assignment from Open Targets Genetics.

Table S4. Association results for previously reported genome-wide signals.

CHR	BP	rsID	Location	Nearest gene	A1*/A2	A1 Freq.	OR (95%)	P	Study+
1	9107187	rs7549256	intronic	*GPR157*	A/C	0.665	0.88 (0.84 -0.93)	$1.79x10^{-6}$	Partanen (2022)
1	77998184	rs4130548	intronic	*DNAJB4*	T/C	0.634	0.95 (0.91-1.00)	0.063	Partanen (2022)
1	150579566	rs16837903	5' UTR	*MCL1*	A/G	0.144	0.81 (0.75-0.87)	$1.31x10^{-8}$	Peljto (2023)
1	214486255	rs4233306	intronic	*PTPN14*	T/C	0.204	0.82 (0.77-0.88)	$1.56x10^{-9}$	Allen (2020)
3	44861942	rs2292181	exonic	*KIF15*	C/G	0.052	1.55 (1.39-1.72)	$1.33x10^{-15}$	Allen (2020)
3	169768483	rs9860874	intronic	*TERC*	A/C	0.276	1.28 (1.22-1.35)	$1.20x10^{-20}$	Fingerlin (2013)
4	88916657	rs2609259	intronic	*FAM13A*	A/C	0.225	1.30 (1.23-1.37)	$1.22x10^{-20}$	Fingerlin (2013)
4	159892716	rs76537958	intergenic	*FSTL5*	A/T	0.966	0.84 (0.74-0.96)	$9.47x10^{-3}$	Partanen (2022)
5	1282299	rs7725218	intronic	*TERT*	A/G	0.324	0.70 (0.67-0.74)	$1.13x10^{-35}$	Mushiroda (2008)
5	169588475	rs116483731	exonic	*SPDL1*	A/G	0.010	2.79 (2.29-3.39)	$1.14x10^{-24}$	Dhindsa (2021)
6	7562999	rs2076295	intronic	*DSP*	T/G	0.530	0.68 (0.65-0.72)	$4.81x10^{-53}$	Fingerlin (2013)
6	43385242	rs1214759	intergenic	6p21.2	A/G	0.321	0.85 (0.81-0.90)	$3.28x10^{-9}$	Allen (2022)
6	35707919	rs9380529	intronic	*FKBP5*	A/G	0.505	0.88 (0.84-0.93)	$4.44x10^{-6}$	Partanen (2022)
7	1829125	rs12537430	intronic	*MAD1L1*	A/G	0.376	0.80 (0.76-0.85)	$4.28x10^{-17}$	Allen (2020)
7	100032719	rs2897075	intronic	*ZKSCAN1*	T/C	0.384	1.28 (1.22-1.35)	$2.97x10^{-23}$	Allen (2022)
7	129095384	rs34288126	intergenic	*TPI1P2*	A/G	0.129	1.12 (1.04-1.20)	$1.63x10^{-3}$	Partanen (2022)
8	119927966	rs10808505	intronic	*DEPTOR*	T/G	0.572	1.20 (1.14-1.26)	$4.78x10^{-13}$	Allen (2020)
9	106717987	rs11788059	intergenic	9q31.2	T/C	0.661	0.86 (0.82-0.91)	$1.12x10^{-8}$	Allen (2022)
10	103881220	rs7100920	3'UTR	*OBFC1*	T/C	0.509	1.17 (1.11-1.23)	$2.70x10^{-10}$	Allen (2022)
10	109470103	rs79684490	intergenic	10q25.1	A/G	0.047	1.29 (1.16-1.43)	$3.10x10^{-6}$	Allen (2022)
11	1219991	rs35705950	intergenic	*MUC5B*	T/G	0.150	5.22 (4.89-5.58)	0#	Seibold (2011)
13	112880670	rs9577395	intronic	*ATP11A*	C/G	0.793	1.27 (1.20-1.36)	$3.91x10^{-14}$	Fingerlin (2013)
15	40424054	rs2304645	intronic	*IVD*	C/G	0.526	1.27 (1.21-1.33)	$1.10x10^{-21}$	Fingerlin (2013)
15	40639510	rs12912339	intronic	*KNL1*	A/G	0.162	1.26 (1.19-1.34)	$4.00x10^{-13}$	Allen (2022)
15	85744679	rs11073517	3'UTR	*AKAP13*	T/C	0.324	1.18 (1.12-1.24)	$2.42x10^{-10}$	Allen (2017)
16	34035	rs367849850	intergenic	*IL9RP3*	G/$	0.042	2.28 (2.03-2.55)	$7.82x10^{-47}$	Donoghue (2023)
16	67895674	rs539683219	intronic	*PSKH1*	TG/T	NA	NA	NA	Partanen (2022)
17	46137449	rs2077551	intronic	*KANSL1* (17q21.31)	C/G	0.788	1.45 (1.36-1.54)	$3.63x10^{-30}$	Fingerlin (2013)
19	4717660	rs12610495	intronic	*DPP9*	A/G	0.694	0.77 (0.73-0.81)	$1.00x10^{-23}$	Fingerlin (2013)
19	5840608	rs708686	intergenic	*FUT6*	T/C	0.281	1.18 (1.12-1.25)	$1.05x10^{-9}$	Partanen (2022)
20	63652817	rs112087793	intronic	*STMN3*	T/C	0.083	0.78 (0.71-0.86)	$2.40x10^{-7}$	Allen (2022)
20	63693038	rs41308092	ncRNA_intronic	*RTEL1*	A/G	0.023	1.62 (1.39-1.89)	$4.70x10^{-10}$	Allen (2022)

*A1: effect allele; OR: Odds Ratio. #p<$5.00x10^{-121}$
$GGGGAGCCTGGAAGCACAC
Coordinates in GRCh38; Freq,: Frequency
+Mushiroda et al 2008 (20); Seibold et al 2011 (21); Fingerlin et al 2013 (4); Allen et al 2017 (6); Allen et al 2020 (10); Dhindsa et al 2021 (22); Allen et al 2022 (23); Partanen et al 2022 (24); Donoghue et al 2023 (8); Peljto et al 2023 (9).

Table S5. Colocalisation analysis between the IPF risk signals and the gene expression eQTL signals in lung, fibroblasts and blood.

Region	Gencode ID (Gene Symbol)	SNP-effect allele (IPF risk allele)	P	NES	Tissue	PP.H0.abf	PP.H1.abf	PP.H2.abf	PP.H3.abf	PP.H4.abf
1q22	ENSG00000160766.14 (*GBAP1*)	rs9426886-T (T)	5.1x10^{-25}	-0.43	Cells cultured fibroblasts	3.62x10^{-39}	1.40x10^{-35}	2.56x10^{-4}	0.988	0.012
	ENSG00000160766.14 (*GBAP1*)	rs9426886-T (T)	2.7x10^{-20}	-0.28	Lung	2.45x10^{-34}	9.44x10^{-31}	2.56x10^{-4}	0.987	0.012
	ENSG00000169231.13 (*THBS3*)	rs9426886-T (T)	4.4x10^{-20}	0.23	Whole blood	2.76x10^{-64}	1.06x10^{-60}	2.58x10^{-4}	0.994	6.02x10^{-3}
	ENSG00000169231.13 (*THBS3*)	rs9426886-T (T)	1x10^{-9}	0.19	Lung	4.28x10^{-21}	1.65x10^{-17}	2.58x10^{-4}	0.996	4.10x10^{-3}
	**ENSG00000185499.16 (*MUC1*)**	**rs9426886-T (T)**	**1.5x10^{-9}**	**0.22**	**Whole blood**	**7.02x10^{-10}**	**2.71x10^{-6}**	**9.96x10^{-5}**	**0.383**	**0.616**
	ENSG00000236263.1 (*RP11-263K19.6*)	rs9426886-T (T)	5.5x10^{-9}	0.13	Whole blood	8.10x10^{-12}	3.12x10^{-8}	2.53x10^{-4}	0.974	0.026
	ENSG00000160766.14 (*GBAP1*)	rs9426886-T (T)	1.8x10^{-8}	-0.21	Whole blood	5.03x10^{-17}	1.94x10^{-13}	2.56x10^{-4}	0.985	0.015
	ENSG00000169241.17 (*SLC50A1*)	rs9426886-T (T)	3.7x10^{-8}	0.13	Cells cultured fibroblasts	8.60x10^{-15}	3.31x10^{-11}	2.57x10^{-4}	0.990	0.010
	ENSG00000231064.7 (*RP11-263K19.4*)	rs9426886-T (T)	2.40x10^{-7}	0.19	Whole blood	2.12x10^{-15}	8.15x10^{-12}	2.58x10^{-4}	0.996	3.89x10^{-3}
	ENSG00000143537.13 (*ADAM15*)	rs9426886-T (T)	9.30x10^{-7}	-0.12	Cells cultured fibroblasts	5.32x10^{-17}	2.05x10^{-13}	1.44x10^{-4}	0.553	0.447
	ENSG00000231064.7 (*RP11-263K19.4*)	rs9426886-T (T)	2.00x10^{-6}	0.14	Lung	2.40x10^{-7}	9.24x10^{-4}	1.41x10^{-4}	0.544	0.455
	ENSG00000225855.6 (*RUSC1-AS1*)	rs9426886-T (T)	2.80x10^{-6}	0.11	Cells cultured fibroblasts	1.29x10^{-15}	4.97x10^{-12}	2.59x10^{-4}	1.000	3.82x10^{-5}
	ENSG00000169242.11 (*EFNA1*)	rs9426886-T (T)	5.50x10^{-5}	-0.12	Lung	1.53x10^{-7}	5.91x10^{-4}	2.50x10^{-4}	0.964	0.035
12q23.1	ENSG00000258343.1 (*RP11-536G4.2*)	rs4762250-G (A)	6.20x10^{-19}	-0.48	Lung	8.63x10^{-73}	1.85x10^{-70}	2.57x10^{-3}	0.549	0.448
	ENSG00000139343.10 (*SNRPF*)	rs4762250-G (A)	3.30x10^{-7}	-0.087	Whole blood	1.01x10^{-6}	2.15x10^{-4}	3.53x10^{-3}	0.754	0.242
	ENSG00000139344.7 (*AMDHD1*)	rs4762250-G (A)	2.10x10^{-5}	-0.18	Lung	1.69x10^{-6}	3.61x10^{-4}	3.21x10^{-3}	0.686	0.310
	**ENSG00000074527.11 (*NTN4*)**	**rs6538677-G (A)**	**4.00x10^{-5}**	**0.13**	**Lung**	**2.88x10^{-4}**	**0.062**	**1.35x10^{-3}**	**0.287**	**0.649**

NES: normalized effect size.

SUPPLEMENTAL FIGURES

Figure S1. Quantile-quantile plot of the observed (y-axis) versus expected -log10 p-values (x-axis) of the meta-analysis results (before genomic control correction): a) autosomal analysis (LDsc inflation factor λ=1.18); b) X chromosome (classic inflation factor λ=1.26).

a)

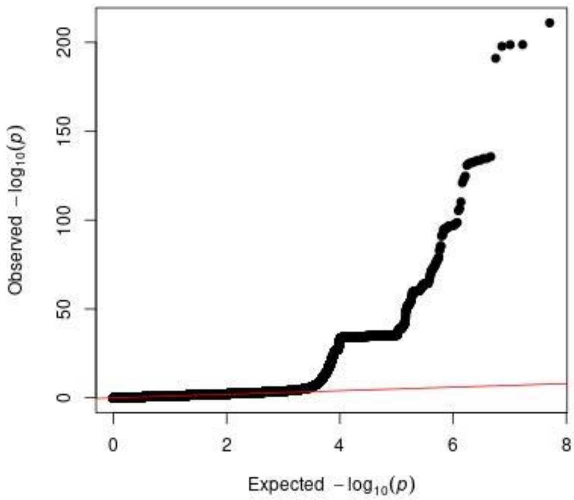

b)

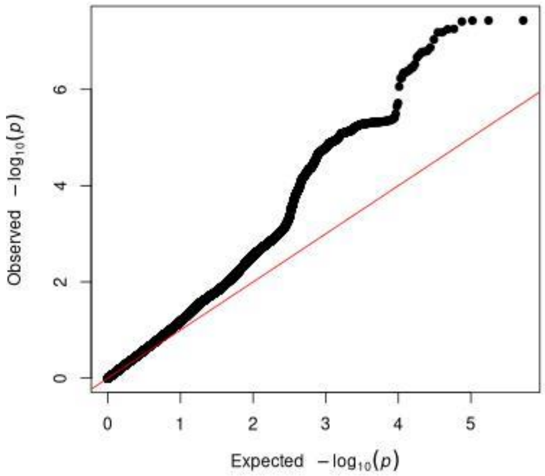

Figure S2. Mirror plots of the signals of interest with the highest posterior probability H4: a) 1q22 region, rs9426886 (intronic to *TRIM46*); and b) 12q23.1 region, rs7957346 (3'UTR to *SNRPF*). The green box represents the gene location.

a) 1q22 region, rs9426886 (intronic to *TRIM46*): *MUC1* in whole blood. IPF association results above and gene expression below.

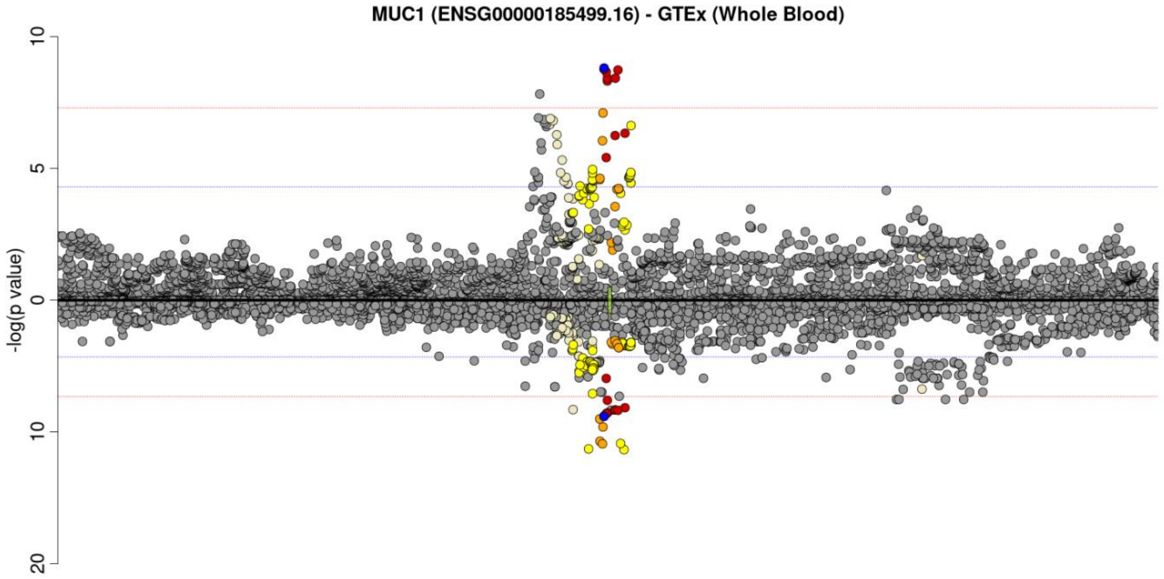

b) 12q23.1 region, rs7957346 (3'UTR to *SNRPF*): *NTN4* in lung. IPF association results above and gene expression below.

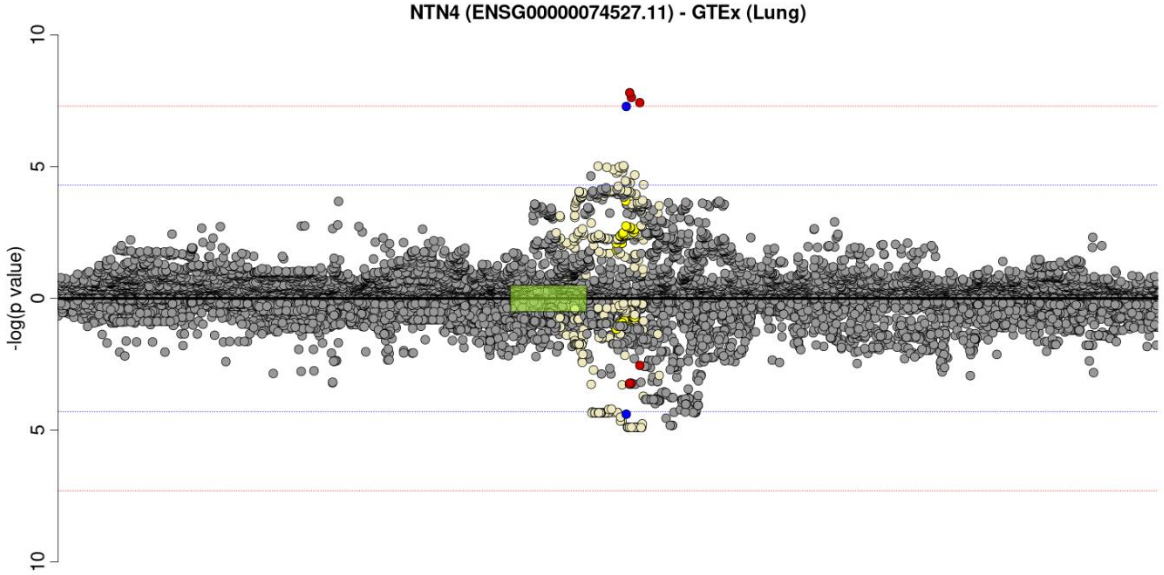

REFERENCES

1. Society ER, American Thoracic Society. American Thoracic Society. American journal of respiratory and critical care medicine. 2002;165(2):277–304.

2. Raghu G, Collard HR, Egan JJ, Martinez FJ, Behr J, Brown KK, et al. An official ATS/ERS/JRS/ALAT statement: idiopathic pulmonary fibrosis: evidence-based guidelines for diagnosis and management. American journal of respiratory and critical care medicine. 2011;183(6):788–824.

3. Raghu G, Remy-Jardin M, Myers JL, Richeldi L, Ryerson CJ, Lederer DJ, et al. Diagnosis of idiopathic pulmonary fibrosis. An official ATS/ERS/JRS/ALAT clinical practice guideline. American journal of respiratory and critical care medicine. 2018;198(5):e44–68.

4. Fingerlin TE, Murphy E, Zhang W, Peljto AL, Brown KK, Steele MP, et al. Genome-wide association study identifies multiple susceptibility loci for pulmonary fibrosis. Nat Genet. 2013;45(6):613–20.

5. Noth I, Zhang Y, Ma S, Flores C, Barber M, Huang Y, et al. Genetic variants associated with idiopathic pulmonary fibrosis susceptibility and mortality: a genome-wide association study. The Lancet respiratory medicine. 2013;1(4):309–17.

6. Allen RJ, Porte J, Braybrooke R, Flores C, Fingerlin TE, Oldham JM, et al. Genetic variants associated with susceptibility to idiopathic pulmonary fibrosis in people of European ancestry: a genome-wide association study. The Lancet respiratory medicine. 2017;5(11):869–80.

7. Reynolds CJ, Sisodia R, Barber C, Moffatt M, Minelli C, De Matteis S, et al. What role for asbestos in idiopathic pulmonary fibrosis? Findings from the IPF job exposures case–control study. Occup Environ Med. 2023;80(2):97–103.

8. Donoghue LJ, Stockwell AD, Neighbors M, Sheng RX, Prabhakaran R, Wolters PJ, et al. Identification of a Genetic Susceptibility Locus for Idiopathic Pulmonary Fibrosis in the 16p Subtelomere Using Whole-Genome Sequencing. American Journal of Respiratory and Critical Care Medicine. 2023;207(7):941–4.

9. Peljto AL, Blumhagen RZ, Walts AD, Cardwell J, Powers J, Corte TJ, et al. Idiopathic pulmonary fibrosis is associated with common genetic variants and limited rare variants. American journal of respiratory and critical care medicine. 2023;207(9):1194–202.

10. Allen RJ, Guillen-Guio B, Oldham JM, Ma S, Dressen A, Paynton ML, et al. Genome-wide association study of susceptibility to idiopathic pulmonary fibrosis. American journal of respiratory and critical care medicine. 2020;201(5):564–74.

11. Martinez FJ, Yow E, Flaherty KR, Snyder LD, Durheim MT, Wisniewski SR, et al. Effect of antimicrobial therapy on respiratory hospitalization or death in adults with idiopathic pulmonary fibrosis: the CleanUP-IPF randomized clinical trial. JAMA. 2021;325(18):1841–51.

12. Chang CC, Chow CC, Tellier LC, Vattikuti S, Purcell SM, Lee JJ. Second-generation PLINK: rising to the challenge of larger and richer datasets. Gigascience. 2015;4(1):s13742–8.

13. Willer CJ, Li Y, Abecasis GR. METAL: fast and efficient meta-analysis of genomewide association scans. Bioinformatics. 2010;26(17):2190–1.

14. Wakefield J. Bayes factors for genome-wide association studies: comparison with P-values. Genetic Epidemiology: The Official Publication of the International Genetic Epidemiology Society. 2009;33(1):79–86.

15. Wang K, Li M, Hakonarson H. ANNOVAR: functional annotation of genetic variants from high-throughput sequencing data. Nucleic Acids Res. 2010;38(16):e164.

16. Giambartolomei C, Vukcevic D, Schadt EE, Franke L, Hingorani AD, Wallace C, et al. Bayesian test for colocalisation between pairs of genetic association studies using summary statistics. PLoS genetics. 2014;10(5):e1004383.

17. Boyle AP, Hong EL, Hariharan M, Cheng Y, Schaub MA, Kasowski M, et al. Annotation of functional variation in personal genomes using RegulomeDB. Genome Res. 2012;22(9):1790–7.

18. Peat G, Jones W, Nuhn M, Marugán JC, Newell W, Dunham I, et al. The open targets post-GWAS analysis pipeline. Bioinformatics. 2020;36(9):2936–7.

19. Biddie SC, Weykopf G, Hird EF, Friman ET, Bickmore WA. DNA-binding factor footprints and enhancer RNAs identify functional non-coding genetic variants. Genome Biol. 2024;25(1):208.

20. Mushiroda T, Wattanapokayakit S, Takahashi A, Nukiwa T, Kudoh S, Ogura T, et al. A genome-wide association study identifies an association of a common variant in TERT with susceptibility to idiopathic pulmonary fibrosis. J Med Genet. 2008;45(10):654–6.

21. Seibold MA, Wise AL, Speer MC, Steele MP, Brown KK, Loyd JE, et al. A common MUC5B promoter polymorphism and pulmonary fibrosis. N Engl J Med. 2011;364(16):1503–12.

22. Dhindsa RS, Mattsson J, Nag A, Wang Q, Wain LV, Allen R, et al. Identification of a missense variant in SPDL1 associated with idiopathic pulmonary fibrosis. Communications biology. 2021;4(1):392.

23. Allen RJ, Stockwell A, Oldham JM, Guillen-Guio B, Schwartz DA, Maher TM, et al. Genome-wide association study across five cohorts identifies five novel loci associated with idiopathic pulmonary fibrosis. Thorax. 2022;77(8):829–33.

24. Partanen JJ, Häppölä P, Zhou W, Lehisto AA, Ainola M, Sutinen E, et al. Leveraging global multi-ancestry meta-analysis in the study of idiopathic pulmonary fibrosis genetics. Cell genomics. 2022;2(10).
